# Supplementary material for: Migratory pattern of zoonotic Toxocara cati and T. canis in experimentally infected pigs
Source: Eur J Clin Microbiol Infect Dis. 2024 Jan 23;43(3):587–96. doi: 10.1007/s10096-024-04753-7 (PMC10917876; doi:10.1007/s10096-024-04753-7)
Supplement: Supplementary file 2 — Supplementary file2 (PDF 761 KB) [file 10096_2024_4753_MOESM2_ESM.pdf]

(0)

(+)

(++)

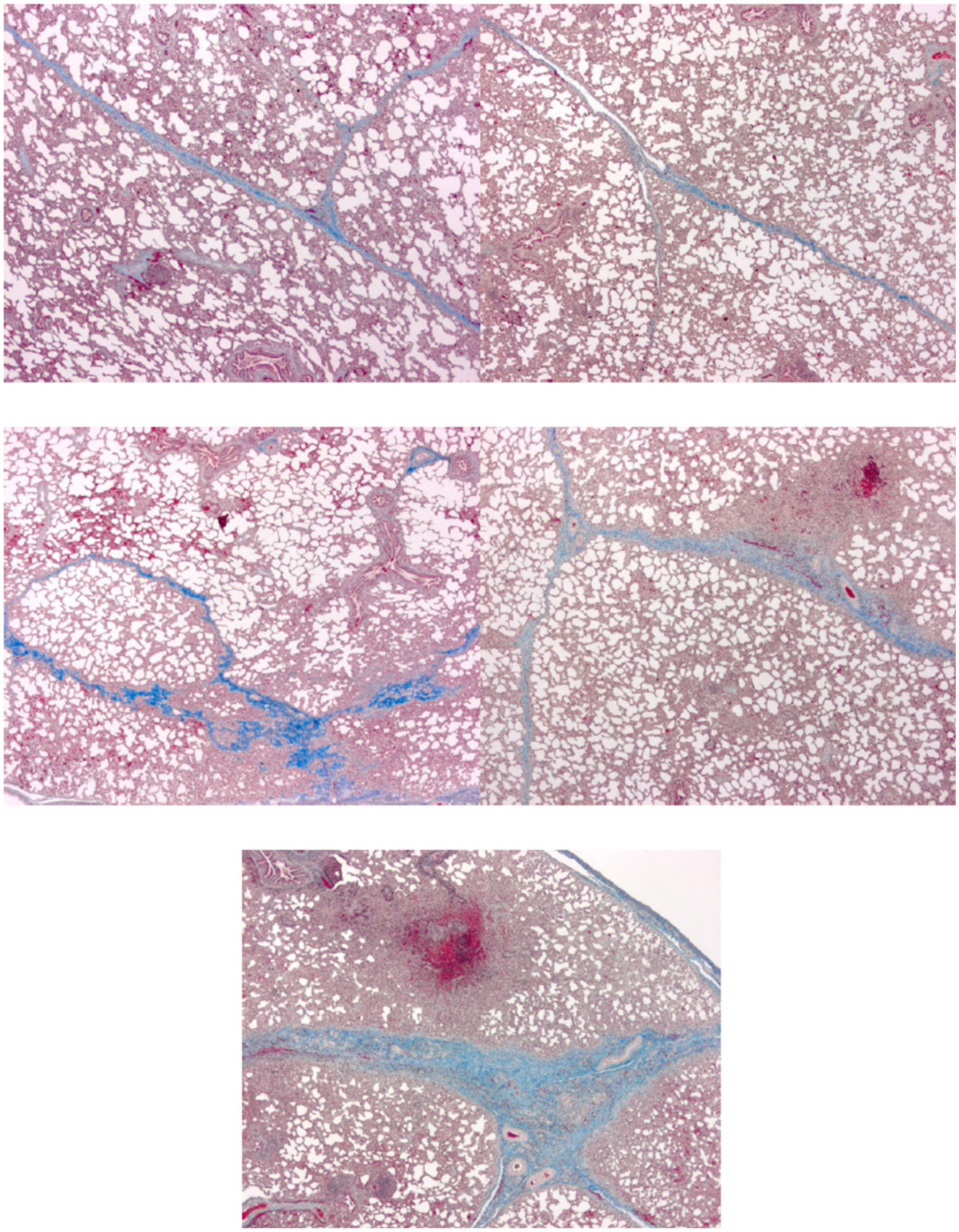

Supplementary Figure 2. Categorization of lung fibrosis (20X): non-existent (0), mild (+), or massive (++). Masson trichrome stains fibrous tissue in the interlobular septa light blue.
