# Supplementary material for: Migratory pattern of zoonotic Toxocara cati and T. canis in experimentally infected pigs
Source: Eur J Clin Microbiol Infect Dis. 2024 Jan 23;43(3):587–96. doi: 10.1007/s10096-024-04753-7 (PMC10917876; doi:10.1007/s10096-024-04753-7)
Supplement: Supplementary file 3 — Supplementary file3 (PDF 455 KB) [file 10096_2024_4753_MOESM3_ESM.pdf]

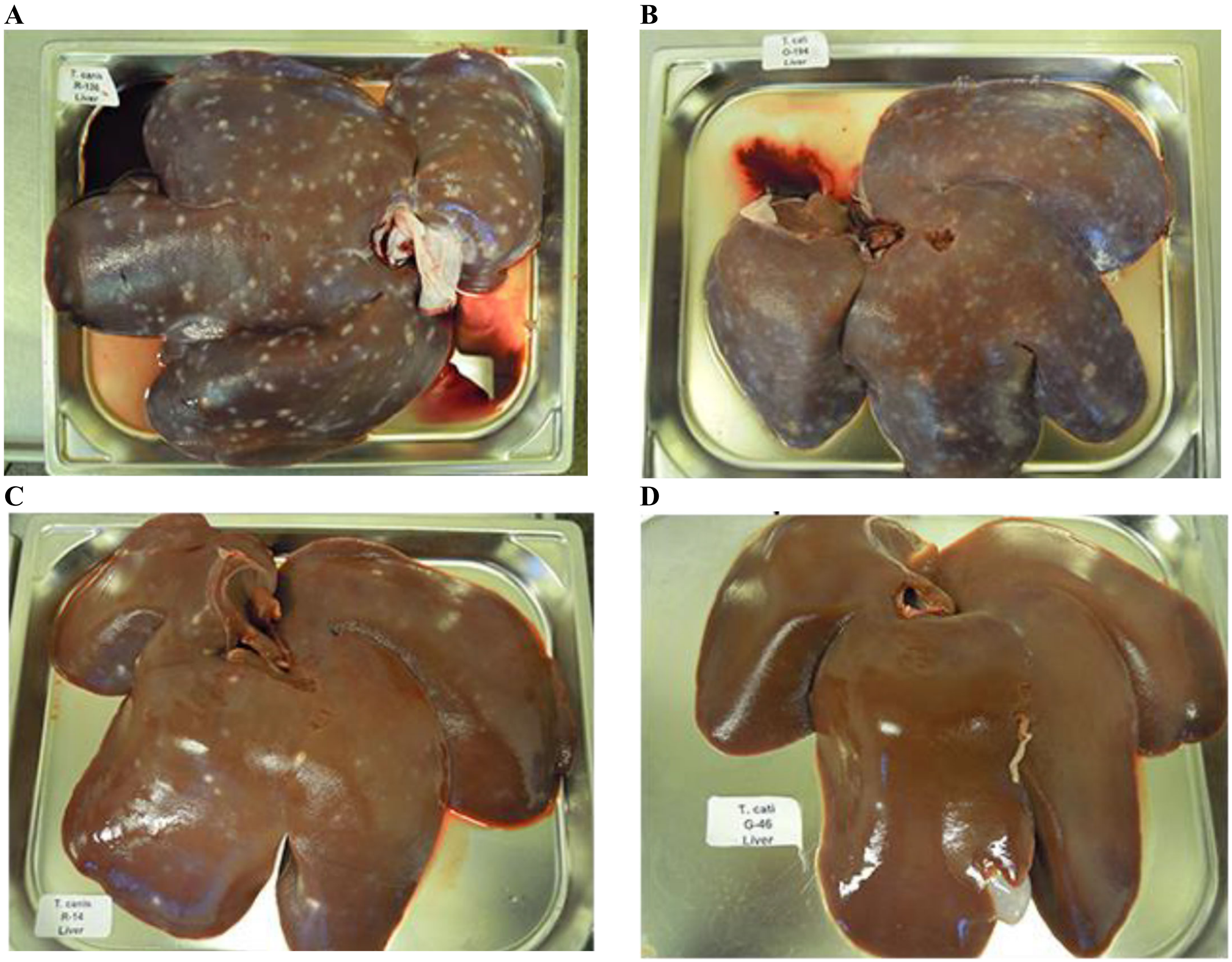

Supplementary Figure 3. Representative livers of pigs infected with *Toxocara* spp. A and B) *T. canis* and *T. cati* infected pigs (50,000 eggs) at day 14 dpi., respectively. C and D) *T. canis* and *T. cati* infected pigs (10,000 eggs) at day 31 dpi., respectively.
