# Supplementary material for: Migratory pattern of zoonotic Toxocara cati and T. canis in experimentally infected pigs
Source: Eur J Clin Microbiol Infect Dis. 2024 Jan 23;43(3):587–96. doi: 10.1007/s10096-024-04753-7 (PMC10917876; doi:10.1007/s10096-024-04753-7)
Supplement: Supplementary file 4 — Supplementary file4 (PDF 88 KB) [file 10096_2024_4753_MOESM4_ESM.pdf]

Supplementary Table 1. Histological evaluation of sections from indicated organs from pigs infected with 50,000 and 10,000 *Toxocara* spp. eggs or uninfected controls on day 14 and 31 days post infection (dpi), respectively

Pig Id	DPI	Infection	Eosinophila			Fibrosis			Necrosis		Granulomas			Emphysema
Scoring system			<25 (0), 25-50 (+), 50-100 (++), >100 (+++)			Nil (0), Moderate (+), Massiv (++)	Nil (0), mild (+), massive (++)	Nil (0), Present (+)	Nil (0), Present (+)		Nil (0), 1-2 (+), 3-4 (++), 5> (+++)			Nil (0), Present (+)
Organ type			Liver	Lung	Lymph node	Liver	Lung	Lymph node	Liver	Lung	Liver	Lung	Lymph node	Lung
42103-14-Y32	14	*T. canis*	+++	+++	+++	++	0	0	+	+	0	++	0	0
42103-1-R136	14	*T. canis*	+++	+++	+++	++	0	0	+	0	0	0	0	0
42103-3-Y33	14	*T. canis*	+++	+++	+++	++	0	0	0	0	0	+	+	0
42103-5-R137	14	*T. canis*	+++	+++	+++	++	+	0	+	+	++	++	0	0
42103-6-Y31	14	*T. canis*	+++	+++	+++	+	0	0	0	0	0	0	0	0
42103-8-R138	14	*T. canis*	++	+++	+++	0	+	+	0	+	0	+	+	0
42103-10-B47	14	*T. cati*	+++	+++	+++	+	+	0	0	+	0	++	0	0
42103-13-O193	14	*T. cati*	+++	+++	+++	0	0	0	0	0	0	0	+	0
42103-4-O195	14	*T. cati*	+++	+++	+++	+	++	0	0	+	0	++	+	0
42103-9-B48	14	*T. cati*	+	+++	+++	0	0	0	0	0	0	+	+	0
42103-2-O194	14	*T. cati*	++	+++	+++	+	+	0	0	0	0	+	+++	0
42103-11-W53	14	Control	0	0	++	0	0	0	0	0	0	0	0	0
42103-12-W56	14	Control	0	0	+	0	0	0	0	0	0	0	0	0
42103-15-W55	14	Control	0	0	+++	0	0	0	0	0	0	0	0	0
42103-16-W57	14	Control	0	0	+	0	0	0	0	0	0	0	0	0
42103-17-W54	14	Control	0	0	+	0	0	0	0	0	0	0	0	+
42103-7-W51	14	Control	0	0	+	0	0	0	0	0	0	0	0	0
41691-13-R18	31	*T. canis*	0	+	+++	0	+	0	0	0	0	0	0	0
41691-15-R20	31	*T. canis*	0	++	+++	+	+	0	0	0	0	+	0	0
41691-1-T15	31	*T. canis*	+++	0	+++	++	0	0	0	0	+++	0	++	+
41691-5-R16	31	*T. canis*	0	0	+++	0	+	0	0	0	0	+	0	0

41691-6-R14	31	*T. canis*	0	+++	+++	0	0	0	0	0	0	0	0	0
41691-7-R19	31	*T. canis*	0	+	+++	0	0	0	0	0	0	0	+	0
41691-8-R12	31	*T. canis*	+++	0	+++	++	0	0	+	0	0	0	0	0
41691-10-G47	31	*T. cati*	0	0	+++	0	0	0	0	0	0	0	+	0
41691-11-G41	31	*T. cati*	0	0	+++	0	+	0	0	0	0	0	++	0
41691-12-G45	31	*T. cati*	0	0	+++	0	0	0	0	0	0	0	+	0
41691-16-G44	31	*T. cati*	0	0	++	0	0	0	0	0	0	0	++	0
41691-18-G43	31	*T. cati*	0	0	+++	0	0	0	0	0	0	0	0	0
41691-2-G42	31	*T. cati*	0	+++	+++	0	+	0	0	0	0	+	0	0
41691-9-G46	31	*T. cati*	0	0	+++	0	0	0	0	0	0	0	0	0
41691-14-R21	31	Control	0	0	++	0	0	0	0	0	0	0	0	0
41691-17-R22	31	Control	$	$	0	$	$	0	$	$	0	$	0	$
41691-3-R24	31	Control	0	0	++	0	0	0	0	0	0	0	0	0
41691-4-R23	31	Control	0	0	++	0	0	0	0	0	0	0	0	0

$ Section not available
